# Clarifying the neural circuit mechanisms of spontaneous social behavior in macaques

**DOI:** 10.3389/fncir.2026.1783133

**Published:** 2026-03-25

**Authors:** Taihei Ninomiya, Takaaki Kaneko, Yuzuha Ono, Kenta Kobayashi, Masaki Isoda

**Affiliations:** 1 Division of Behavioral Development, Department of System Neuroscience, National Institute for Physiological Sciences, National Institutes of Natural Sciences, Okazaki, Aichi, Japan; 2 Department of Physiological Sciences, School of Life Science, The Graduate University for Advanced Studies, SOKENDAI, Hayama, Kanagawa, Japan; 3 Center for Postgraduate Clinical Training and Career Development, Nagoya University Hospital, Nagoya, Aichi, Japan; 4 Section of Viral Vector Development, Center for Genetic Analysis of Behavior, National Institute for Physiological Sciences, National Institutes of Natural Sciences, Okazaki, Aichi, Japan

**Keywords:** amygdala, freely moving macaques, nucleus accumbens, pathway-specific manipulation, viral vectors

## Abstract

Research using nonhuman primates has investigated how the brain processes and represents a wide range of socially relevant information, such as others’ faces, actions and rewards. While our understanding has expanded considerably in recent years, much of the research has been conducted under highly controlled task conditions, leaving the neural underpinnings of naturally occurring social behaviors largely unexplored. In this Perspective, we first highlight recent efforts utilizing freely behaving primates to overcome these challenges. We then detail our own experiments, demonstrating how the combined use of behavioral analysis and neural manipulation techniques in freely moving macaques enabled us to identify a specific neural circuit critical for the spontaneous expression of mounting behavior. These strategies offer novel opportunities to validate and extend established knowledge concerning the neural basis of social behavior in experimental settings that more closely resemble those occurring in a real world.

## Introduction

Social behavior is a cornerstone of survival, reproduction, and group cohesion across species. Considerable effort has been directed toward understanding the neural mechanisms underlying social behavior and its related social cognitive functions. Disruptions in social cognitive functions—and the resulting abnormalities in social behavior—are central to numerous neurodevelopmental disorders, such as autism spectrum disorder (ASD) and attention-deficit/hyperactivity disorder, making the investigation of their neural substrates both scientifically and clinically important.

Nonhuman primates, especially macaques, have been used as a model for studying social cognition and behavior. Macaques acquire social information by observing others’ eye gaze and facial expressions, and exhibit a rich repertoire of social behaviors in both natural and laboratory environments (Emery et al., 1997; Chang et al., 2013; Nakamichi, 2016; Hayashi et al., 2020), making them well suited for social neuroscience research. For example, pioneering work by Perrett et al., (1985, 1987, 1990, 1992) demonstrated that neurons in the temporal cortex process key information for social cognition. These findings revealed specialized neural mechanisms for processing socially relevant visual features. For decades, however, investigations into the neural underpinnings of social cognitive functions in nonhuman primates have predominantly relied on controlled tasks, often using simplified stimuli such as static images of facial expressions.

Social neuroscience research in nonhuman primates has extended to include more interactive paradigms. A variety of experimental settings have been developed to study social cognitive functions. Indeed, ecological contexts vary along a continuum rather than a binary property, and various situations—from highly controlled laboratory tasks to dynamic face-to-face interactions—can be positioned along this spectrum [for review, see Fan et al. (2021)]. These settings have been successful in capturing various aspects of social behaviors, including monitoring (Falcone et al., 2016; Noritake et al., 2018; Grabenhorst et al., 2019; Dal Monte et al., 2020; Ninomiya et al., 2020), cooperation (Haroush and Williams, 2015; Visco-Comandini et al., 2015) and competition (Hosokawa and Watanabe, 2012; Ballesta et al., 2014), thereby allowing us to investigate their neural underpinnings at the single-neuron level. Real-time, face-to-face interactive paradigms and experimental settings are still actively developed and refined (Moeller et al., 2023; Isbaner et al., 2025). Alongside human neuroimaging studies, this body of work has revealed that the primate brain is equipped with specialized networks for processing social information, often referred to as the “social brain” (Frith, 2007; Adolphs, 2009; Van Overwalle and Baetens, 2009). These insights laid the groundwork for subsequent investigations into how social information is represented in the primate brain. Despite these advances, the full complexity of naturally occurring social behaviors and their neural correlates remains largely unexplored. This is because interactive paradigms to date have been developed within structured task frameworks that constrain the range of observable behaviors.

As our understanding of brain functions has grown considerably in controlled environments, a reasonable next step is to extend this knowledge to more complex situations, such as free-moving conditions where animals can interact with others and environments with negligible constraints, if any. A growing number of studies show that neural encoding in the brain can differ between strictly controlled stimuli under restricted conditions and naturalistic stimuli under freely moving conditions (Jovanovic et al., 2022; Lanzarini et al., 2025). Similarly, the neural encoding can, in some contexts, be better modeled by naturalistic stimuli. For example, the decoding model trained to predict neural activity under a naturalistic condition can accurately predict neural activity under a strictly controlled condition, whereas the opposite is not the case (Talebi and Baker, 2012; Lanzarini et al., 2025). Naturalistic behavior is essential to understand the neural dynamics in the ecological context where the brain originally operates.

### Technological advancements: enabling investigation in freely moving animals

Recent technological advances have expanded the potential to study the neural basis of social cognition in freely moving animals. Wireless neural recording using miniature loggers has become widely available in rodents, and its application is extending to use of larger animals, enabling chronic, untethered monitoring of brain activity during natural behavior (Danjo et al., 2018; Omer et al., 2018; Mao et al., 2021; Rose et al., 2021).

An obvious challenge in analyzing freely moving animals is how to process a rich variety of behavioral repertoires inherent in such environments. Deep learning-based pose estimation and action recognition systems (Mathis et al., 2018; Bala et al., 2020; Labuguen et al., 2020; Kaneko et al., 2024; Matsumoto et al., 2025) can now automatically detect and quantify complex behavioral patterns with accuracy and efficiency exceeding human annotators. Traditional manual scoring has been largely replaced by sophisticated algorithms capable of detecting and quantifying subtle behavioral changes across extended observation periods.

Recent studies employing wireless neural recording and markerless tracking in freely moving primates have enabled unprecedented access to naturalistic social interactions, spatial navigation, and decision-making processes (Franch et al., 2024; Testard et al., 2024; Lanzarini et al., 2025). These innovations allow researchers to capture dynamic, context-dependent neural activity during spontaneous behavior. Collectively, these approaches lay the groundwork for a more ecologically valid social neuroscience, bridging controlled experimentation with the complexity of real-world behavior.

In addition to the technological advancements mentioned above, the causal manipulation of neuronal activity in the primate brain has been extensively developed, yielding invaluable insights into the neural mechanisms of sensory, motor, and cognitive functions (Kinoshita et al., 2012; Gerits and Vanduffel, 2013; Inoue et al., 2015; El-Shamayleh et al., 2016; Nagai et al., 2020; Ninomiya et al., 2020; Oyama et al., 2022; Noritake et al., 2023). The combination of these methodologies allows researchers to investigate the functions of neural circuits from both correlational and causal perspectives during naturalistic social interactions.

### An example: causal manipulation of the amygdala-nucleus accumbens circuit

**Figure 1:**
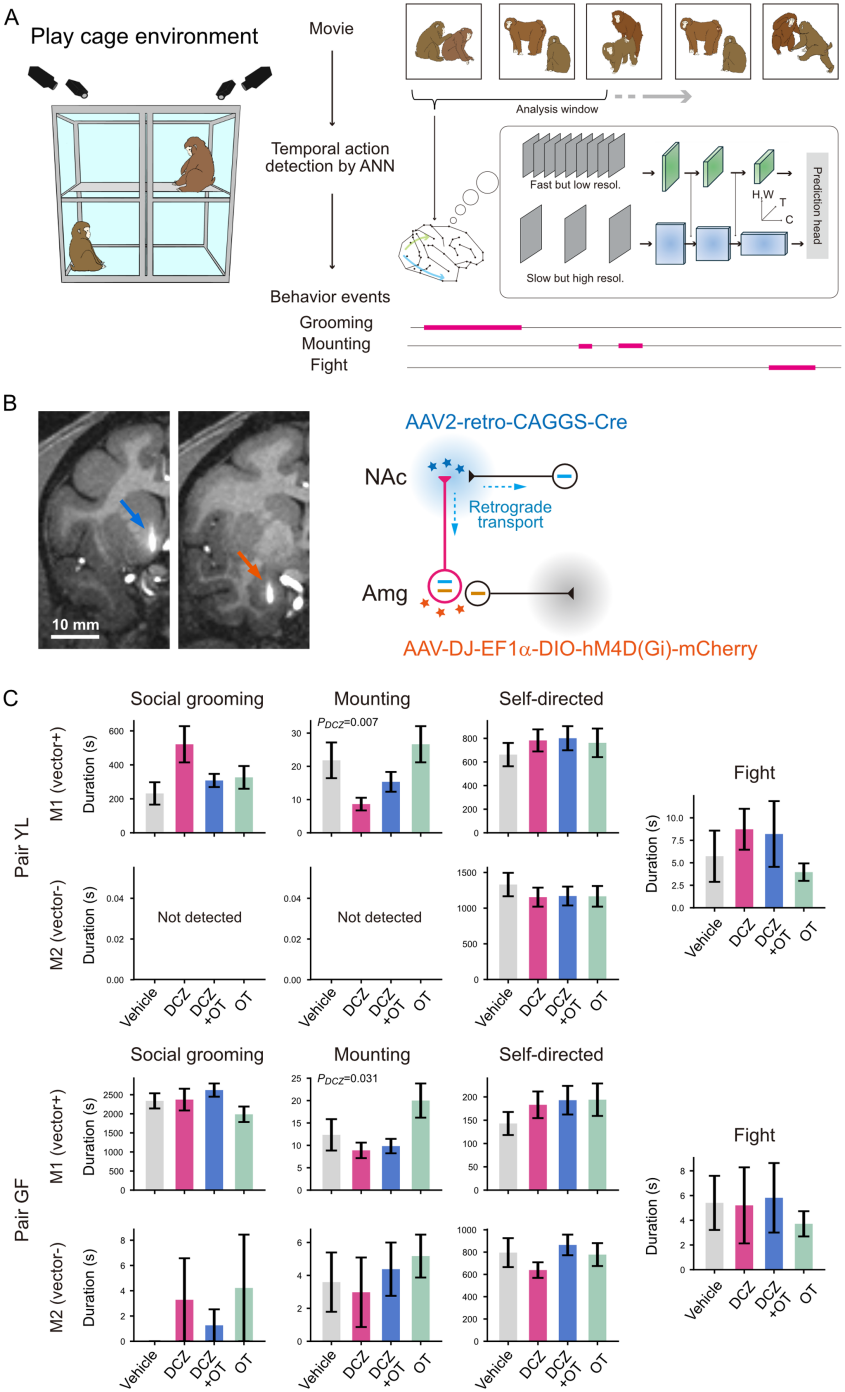
Causal evaluation of the AMG-to-NAc pathway in social behavior of freely interacting macaque pairs. **(A)** Schematics of the play cage environment and behavioral analysis using an ANN model. The ANN, implemented with two streams for visual analysis as the backbone (Feichtenhofer et al., 2019) and with a transformer architecture in the prediction head (Zhang et al., 2022), was used to predict the onsets and offsets of behavioral events. The model was pre-trained on datasets comprising 260k video clips with annotations for 400 variations of human actions (Kay et al., 2017) and fine-tuned using in-house annotations of monkey behaviors, following an end-to-end training method designed for fine-tuning of massive video-recognition models (Zhao et al., 2023). H, W: height and width for spatial dimension, T: temporal dimension, C: feature size. **(B)** Pathway-selective blockade using double viral vector technique. Retrograde and anterograde vectors were injected into the NAc and AMG, respectively (left). The injection sites were visualized by co-injecting Gadoteridol and vectors (arrows). Only NAc-projecting neurons in the AMG are double-infected, thereby expressing inhibitory DREADD (right). **(C)** Effect of pathway blockade and OT inhalation on social and self-directed behaviors. In the DCZ + OT condition, OT was administered only to M1. The performance of social grooming and mounting was attributed to the monkey acting as the groomer and the mounter, respectively. Self-directed behaviors include self-scratch, self-grooming, and manipulation of a neck collar. Fight is an agonistic interaction between two animals. The detection accuracy, as indexed by F1-score, was over 0.8 on average. The bars and errors show the mean and s.e.m. of multiple sessions from two pairs of monkeys (12 sessions × 4 conditions for each pair). Statistical tests were performed using a linear mixed model, with DCZ and OT treatments as fixed effects and pair as a random effect. The statistical significance of the fixed effects was estimated using the Wald test. **p < 0.01.

Having outlined the recent methodological advances, we now turn to a specific experiment from our own work that exemplifies their combined strength. By combing a free-moving environment and circuit manipulation techniques, we sought to probe the circuit basis for the expression of social behavior. In contrast to the difficulty in investigating such aspects of behavior in conventional, controlled settings, free-moving animals often show various social behaviors spontaneously. Here, we employed a dual viral vector infection technique to express an inhibitory designer receptor exclusively activated by designer drug (DREADD) (Roth, 2016) for causal circuit manipulations. The intervention can be induced by administrating deschloroclozapine (DCZ), a highly selective, potent, and metabolically stable DREADD agonist (Nagai et al., 2020). We also tested whether intervention-induced behavioral effect, if any, can be mitigated by inhalation of oxytocin (OT), a potential therapeutic approach for ASD (Andari et al., 2010; Gordon et al., 2013). Using this chemogenetic approach in conjunction with OT administration, we examined the possible involvement of the amygdala-to-nucleus accumbens (Amg-to-NAc) pathway—a circuit implicated in ASD (Yahata et al., 2016)—in the expression of social behavior in freely interacting male macaque pairs (Figure 1).

Four male macaques (*Macaca fuscata*) were used in this study. Monkey Y (age 9, 10.4 kg) was paired with monkey L (age 7, 10.2 kg), and monkey G (age 6, 9.8 kg) was paired with monkey F (age 6, 8.7 kg). All animal care and experimentation protocols were approved by the Institutional Animal Care and Use Committee of the National Institutes of Natural Sciences, and were conducted in accordance with the Guidelines for the Care and Use of Nonhuman Primates in Neuroscience Research of the Japan Neuroscience Society. The two pairs of monkeys were initially habituated to a play cage (height, 1775 mm; width, 1610 mm; depth, 830 mm) designed for neuroethological experiments (Figure 1A, left). Video data were collected during this period to optimize an artificial neural network (ANN) for the automated behavioral analysis (Figure 1A, right). After habituation, one individual from each pair was selected for viral vector injection into the NAc (AAV2 retro-CAGGS-Cre) and Amg (AAV DJ-EF1a-DIO-hM4D(Gi)-mCherry) (Figure 1B) in both hemispheres. Based on daily observations, the two individuals chosen for vector injection were dominant in the hierarchy. Note that the dominant individuals were chosen not because of any assumptions regarding the manipulation effects in relation to the dominance, but rather to maintain consistency across pairs. The coordinates of the injection tracks were determined based on MR images. The vector injections were made stereotaxically into two sites for each brain region. The viral vector was deposited at one or two different depths for each site in the NAc and Amg, respectively (0.8–1.0 μL at each depth). The monkeys that received viral injections were referred to as M1 and their partners as M2. The animals were then placed in the play cage two to three times per week over the course of approximately 1 month for viral expression and re-habituation, following a postoperative recovery period. The main experiment, consisting of two factors (DCZ and OT), was then initiated. Regarding the DCZ administration, DCZ (0.1 mg/kg) dissolved in sulfoxide (DMSO) with saline was administered intramuscularly to inhibit the target pathway. As a vehicle control condition, DMSO diluted with saline was administered intramuscularly. The OT inhalation was performed when M1 was fully awake, immediately after the DCZ or vehicle injection. After stabilizing M1’s head, OT (10 IU/mL; Zenoaq) was delivered via nebulization (Pari Baby Nebulizer) into the nose and mouth continuously for 15 min. Nebulized distilled water served as a control. In any conditions, the subject entered the play cage at least 10 min after DCZ or vehicle injections. To avoid possible carryover effects between conditions, recording sessions were spaced at least 1 day apart and limited to a maximum of three sessions per week. Each session lasted 2–3 h, and was repeated 12 times for each condition in each pair. A number of behavioral repertoires were automatically classified by the ANN and compared across the experimental conditions.

We found that the expression of mounting behavior by M1 onto M2 (i.e., M1 as a mounter and M2 as a mountee) was significantly decreased following the pathway blockade in each pair (Figure 1C; *p* = 0.007 for pair YL and *p* = 0.031 for pair GF; two-way ANOVA; main effect of DCZ). Interestingly, no significant effects were observed in either pair for other social and nonsocial behaviors (grooming, *p* = 0.34 for pair YL and *p* = 0.069 for pair GF; self-directed, *p* = 0.43 for pair YL and *p* = 0.44 for pair GF; fight, *p* = 0.20 for pair YL and *p* = 0.62 for pair GF). The duration of M1’s mounting increased after OT inhalation; however, this effect was not statistically significant either as the main factor (*p* = 0.35 for pair YL and *p* = 0.064 for pair GF) or interaction (*p* = 0.27 for pair YL and *p* = 0.834 for pair GF). On the other hand, no significant effect was observed in M2’s behaviors in each pair for the DCZ administration (grooming, not detected for pair YL and *p* = 0.70 for pair GF; mounting, not detected for pair YL and *p* = 0.68 for pair GF; self-directed, *p* = 0.60 for pair YL and *p* = 0.80 for pair GF) or OT inhalation (grooming, not detected for pair YL and *p* = 0.85 for pair GF; mounting, not detected for pair YL and *p* = 0.40 for pair GF; self-directed, *p* = 0.69 for pair YL and *p* = 0.15 for pair GF). Note that we did not distinguish which individual (M1 or M2) initiated the fight behavior; however, the statistical results described above were assigned to M1 for convenience.

Since the entire experiment spanned several months, the behavioral expression may have changed over the course of the experiment by other factors such as the relationship within each pair. While this is unlikely because the four conditions conducted in the main experiment (i.e., vehicle, DCZ, OT, DCZ + OT) were counterbalanced during the experimental period, we also examined the temporal aspect of the behavioral data by applying a spline model fitting to the social and non-social behavior data across sessions. By comparing the residuals obtained from the same model across the conditions, we were able to evaluate differences in condition effects while controlling for time-dependent influences. A significant discrepancy was observed in the fitted residuals only for the DCZ condition (pair YL, *p* = 0.0036; pair GF, *p* = 0.023; Wald test), suggesting that any effects over time cannot account for the changes observed following the circuit manipulation.

Prior to the main experiment, we also conducted a control experiment for pair GF to examine any potential side effects of the DCZ administration. DCZ or vehicle was administered to M1 in the same manner as described above, but before the viral vector injections. We found no significant differences in social or self-directed behaviors between the DCZ and vehicle injections for M1 (grooming, *p* = 0.43; mounting, *p* = 0.40; self-directed, *p* = 0.32; fight, *p* = 0.77; *n* = 8) or M2 (grooming, not detected; mounting, not detected; self-directed, *p* = 0.61), which ruled out the possibility that the behavioral changes observed in the main experiment were due to the side effect of DCZ itself.

**Figure 2:**
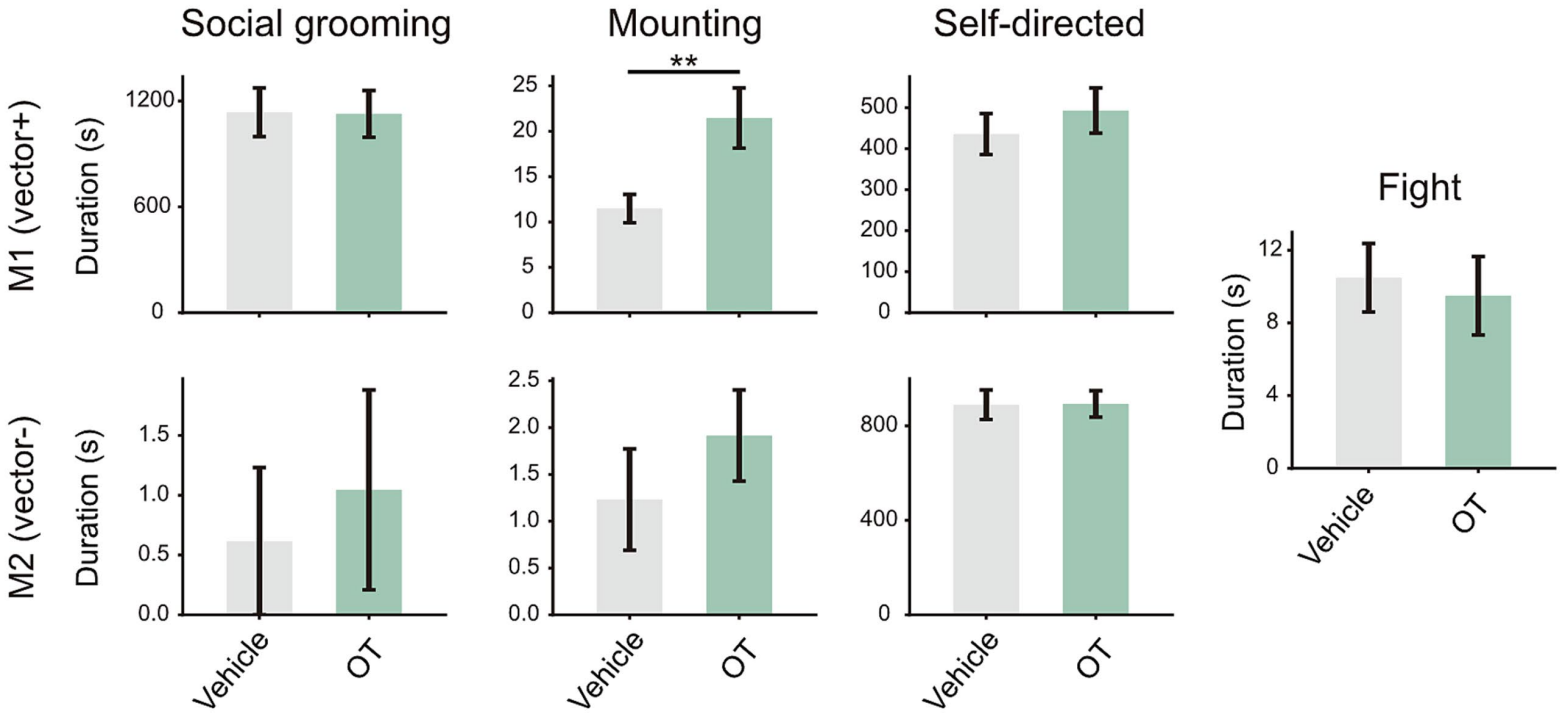
Effect of OT inhalation on social and self-directed behaviors examined outside the context of pathway blockade. In the OT condition, intranasal OT was administered to either M1 or M2. Data derived from OT administration to M1 and M2 were pooled for analysis. A total of 80 sessions were collected from four individuals. One session was excluded from the analysis due to a technical failure. Other conventions are as in Figure 1C.

To assess an independent effect of OT on various social and nonsocial behaviors, we quantified the total duration of occurrence for each behavior in all four individuals (Figure 2). Compared to distilled water inhalation, OT inhalation led to a significant increase in M1’s mounting behavior (Wald test; *p* = 0.008), irrespective of whether inhaled OT was administered to M1 or M2. M2 also showed a longer duration for the mounting behavior, but this effect was not statistically significant (Wald test; *p* = 0.36). The individual difference in the OT effect might be ascribed to hierarchical status, with M1 being dominant over M2.

These findings revealed that the Amg-to-NAc pathway plays a role in expressing specific social behaviors. While social behaviors are complex and their meanings are largely context-dependent, mounting and grooming are generally thought to serve different functional roles. Mounting carries distinct social meanings, such as asserting dominance in hierarchical contexts and mitigating conflict to reduce aggression or signal submission, thereby acting as a tension diffuser (Reinhardt et al., 1986; Faraut et al., 2015; Anzá et al., 2021; Clive et al., 2023). Grooming, which was not affected by the pathway manipulation in our study, fosters long-term social bonds, reduces anxiety, and promotes group cohesion (Ueno and Nakamichi, 2018; Disarbois and Duhamel, 2024). The selective reduction in mounting (but not grooming), along with its possible mitigation by OT inhalation, suggests that the Amg-to-NAc pathway plays a causal role in behavioral expression related to dominance, submission, or social tension regulation, under the influence of oxytocinergic regulation.

## Discussion

Our approach illustrates the power of pathway-specific manipulation in freely moving macaques. The behavioral effects emerged in the naturalistic context and included spontaneous behaviors that are difficult to elicit in conventional task settings. It should be emphasized, however, that while naturalistic paradigms offer unparalleled access to spontaneous and highly contextual social behaviors, controlled experiments remain essential for dissecting detailed neural mechanisms for social information processing. We therefore propose that the integration of unconstrained and controlled approaches represents a novel direction for systems neuroscience studying social cognition and behavior. For example, this integrated framework aligns well with the phenotype-driven approach (Isoda et al., 2018; Ninomiya et al., 2022), enabling detailed investigation of spontaneously occurring behavioral abnormalities that may have been unnoticed. Such an approach facilitates more comprehensive evaluation of potential disease models and their translational relevance.

Recent findings have revealed a critical dimension that extends beyond simple complementarity: neural coding strategies may differ qualitatively across unconstrained versus controlled contexts (Lanzarini et al., 2025). These systematic discrepancies highlight a key issue in systems neuroscience regarding neural ecological validity, that is, whether laboratory paradigms engage the same neural mechanisms as natural contexts. Rather than problematic, such divergences are informative: they reveal context-dependent dynamics and hidden variables that modulate circuit function. This reframes the relationship between controlled and unconstrained approaches—not merely as complementary, but as mutually validating. Naturalistic observations can guide hypothesis generation by revealing emergent patterns and hidden variables, while controlled paradigms allow for systematic testing and mechanistic dissection. A powerful framework would involve long-term tracking of neural populations across both contexts, enabling iterative cycles of discovery, validation, and ecological confirmation. While the current study demonstrates the feasibility of pathway manipulation in unconstrained settings, the full potential of this approach will be realized through long-term tracking, which is becoming increasingly feasible with ongoing technological advances (Feingold et al., 2012; Koyano et al., 2023). Social behaviors are not static; they evolve through repeated interactions, social learning, and developmental processes. Short-term interventions capture immediate circuit effects, but they may miss critical dynamics that emerge over weeks, months, or even years.

Longitudinal tracking of individual neurons provides several key insights. For example, it allows us to distinguish between transient and persistent behavioral changes following circuit manipulation. Long-term studies can reveal how neural circuits are reorganized in response to chronic manipulation or environmental changes. Such plasticity mechanisms are particularly relevant for understanding therapeutic interventions, as their goals are often expected to have sustained effects. Also, longitudinal designs are essential for capturing developmental trajectories. Social cognitive abilities in primates, like humans, mature over extended periods. While technical difficulties still exist, tracking neural activity and behavior across developmental stages, from juvenile to adult, would reveal how social circuits are shaped by experience and how disruptions at different developmental windows lead to distinct behavioral phenotypes. This temporal dimension is especially critical for modeling neurodevelopmental disorders, where early interventions can have profound long-term consequences (Dawson et al., 2010; Zwaigenbaum et al., 2015). The integration of wireless recording systems with automated behavioral analysis makes such longitudinal studies increasingly feasible.

Theoretical frameworks will become increasingly important, as the integration of these technologies yields rich, high-dimensional datasets. These frameworks offer computational models that can generate testable predictions and help interpret results, thereby guiding future experiments and bridging the gap between neural mechanisms and complex social behaviors. The potential rewards are substantial: a comprehensive understanding of social cognition that spans from single neurons to complex behaviors, from milliseconds to months, and from controlled laboratory settings to more naturalistic environments.

## Data Availability

The raw data supporting the conclusions of this article will be made available by the authors, without undue reservation.
